# Nano zero-valent iron loaded corn-straw biochar for efficient removal of hexavalent chromium: remediation performance and interfacial chemical behaviour

**DOI:** 10.1039/d2ra04650d

**Published:** 2022-10-05

**Authors:** Yuzhen Wei, Run Chu, Qinhu Zhang, Muhammad Usman, Fasih Ullah Haider, Liqun Cai

**Affiliations:** College of Forestry, Gansu Agricultural University Lanzhou 730070 P. R. China cailq@gsau.edu.cn; College of Resources and Environmental Sciences, Gansu Agricultural University Lanzhou 730070 P. R. China; Gansu Provincial Key Laboratory of Arid Land Crop Science, Gansu Agricultural University Lanzhou 730070 P. R. China; PEIE Research Chair for the Development of Industrial Estates and Free Zones, Centre for Environmental Studies and Research, Sultan Qaboos University Al-Khoud 123 Muscat Oman

## Abstract

To improve the poor stability of nano zero-valent iron (nZVI), corn-straw biochar (BC) was used as a support for the synthesis of composites of nZVI-biochar (nZVI/BC) in different mass ratios. After a thorough characterization, the obtained nZVI/BC composite was used to remove hexavalent chromium [Cr(vi)] in an aquatic system under varying conditions including composite amount, Cr(vi) concentration, and pH. The obtained results show that the treatment efficiency varied in the following order: nZVI–BC (1 : 3) > nZVI–BC (1 : 5) > nZVI alone > BC alone. This order indicates the higher efficiency of composite material and the positive effect of nZVI content in the composite. Similarly, the composite dosage and Cr(vi) concentration had significant effects on the removal performance and 2 g L^−1^ and 6 g L^−1^ were considered to be the optimum dose at a Cr(vi) concentration of 20 mg L^−1^ and 100 mg L^−1^, respectively. The removal efficiency was maximum (100%) at pH 2 whereas solution pH increased significantly after the reaction (from 2 to 4.13). The removal kinetics of Cr(vi) was described by a pseudo-second-order model which indicated that the removal process was mainly controlled by the rate of chemical adsorption. The thermodynamics was more in line with the Freundlich model which indicated that the removal was multi-molecular layer adsorption. TEM-EDS, XRD, and XPS were applied to characterize the crystal lattice and structural changes of the material to specify the interfacial chemical behaviour on the agent surface. These techniques demonstrate that the underlying mechanisms of Cr(vi) removal include adsorption, chemical reduction–oxidation reaction, and co-precipitation on the surface of the nZVI–BC composite. The results indicated that the corn-straw BC as a carrier material highly improved Cr(vi) removal performance of nZVI and offered better utilization of the corn straw.

## Abstract 2

Loading nano zero-valent iron (nZVI) on corn-straw biochar improved the stability of nZVI and the removal of Cr(vi) in an aqueous system. There happened adsorption, reduction–oxidation and co-precipitation on the composite surface in removal process.

## Graphical abstract

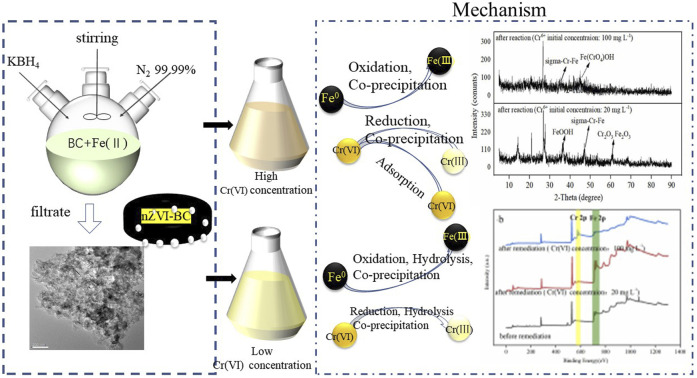

## Introduction

Due to human activities and rapid industrialization such as chemical production, smelting, mining, metallurgy, and pharmaceutical production,^1^ heavy metals (Hg, Cd, Cr, Pb, As) are frequently encountered in water, soil, and other environmental matrices and, thus, have become a major global environmental problem.^2^ Heavy metals (HMs) not only damage the growth of plants and animals irreversibly but also enter the human body through enrichment of the food chain, causing various diseases.^3,4^ Among these HMs, chromium (Cr) is a transition element and is the seventh most abundant metal in the Earth's crust.^5^ Chromium often exists in trivalent (Cr(iii)) and hexavalent states (Cr(vi)). Because trivalent chromium [Cr(iii)] is less toxic than Cr(vi), Cr(vi) is considered as a primary contaminant due to its high toxicity, carcinogenicity and other widespread health concerns.^6,7^ Thus, it is necessary and urgent to remove Cr(vi) from the environment efficiently and safely. Many strategies have been developed for Cr(vi) removal from wastewater, soil, and other environmental media, including chemical reduction, precipitation, adsorption, ion exchange, photocatalysis, biodegradation, *etc.*^8–10^. A large number of low-cost materials have been employed to seek an effective agent for Cr(vi) removal in recent studies such as carbon materials, minerals, organic or biological materials, agricultural wastes, polymeric materials, and so on.^11,12^

Currently, nanomaterials, particularly nano zero-valent iron (nZVI) have shown viable potential to remove various pollutants such as organic contaminants,^13,14^ HMs,^15,16^ nitrate-nitrogen,^17^ and phosphate.^18^ However, because of its poor chemical stability, the nZVI is easily oxidized by oxygen and agglomerates due to its magnetism, which leads to lower chemical activity and limits its wide application.^14^ To solve these problems, many strategies have been employed in literature. Mostly, physico-chemical strategies are used to modify nZVI by introducing new elements or chemicals during its synthesis or by its loading on other materials^15,19–21^ such as carbon,^22^ montmorillonite microparticles,^19^ attapulgite clays,^13^ and so on. Of all these materials, biochar (BC) has shown an excellent performance.^23^ BC is a porous carbon-rich material produced by anaerobic pyrolysis of biomass and has a strong stability.^24,25^ Because of its large specific surface area, well-developed pore structure, ion exchange capacity, and rich surface functional groups such as carboxyl, phenolic hydroxyl, carbonyl, and quinonyl functional groups, BC is no longer only confined to the promotion of crop growth and productivity but has been applied for the remediation of contaminated environments.^26–28^ Therefore, BC has become a research hotspot in recent years and one of the best modifying materials to remove potentially toxic elements.^27,29^ Many studies have been reported to remove HMs by loading pure nZVI or modified nZVI on BC.^30–32^ However, the HMs remediation performance varies with the raw material that BC is synthesized from ref. 33. The BC synthesized from lignocellulose,^34^ chicken manure,^35^ fruit shell,^36^ crop residues,^37^ and other materials has different element composition, density, porosity, and pH. All these factors affect the surface functional groups and lead to a wide divergence of HMs remediation.

Corn-straw is an agricultural waste which is produced in huge quantities of about 303 million tons annually around the world. The USA has the highest yield (140.86 million tons) followed by Asia (33.90 million tons), Europe (28.61 million tons) and Oceania (0.24 million tons).^38^ In developing countries, most of these wastes are burnt on farms after harvest or landfilling, not only generating large amounts of greenhouse gases and air pollution, but also causing huge energy loss, so these wastes are labeled ‘agro-losses’.^39^ One of the best ways to reduce the losses and solve the problem is by using them as feedstock to produce bio-based materials. Moreover, as mentioned above, the removal performance of the loaded nZVI is greatly affected by factors like the nature of biochar support, which is dictated by the raw materials of biochar, and iron contents. Therefore, it is necessary to determine the feasibility of using corn-straw biochar as a support for nZVI for its use to remove Cr(vi) and identify the treatment efficiency, optimum conditions and the underlying reaction mechanisms. Moreover, the conversion of agricultural wastes to biochar is of great significance regarding the economic and environmental aspects.^40^

Here, we propose a hypothesis that the corn-straw biochar can highly improve the poor stability of nZVI and get a better performance in modifying nZVI for Cr(vi) remediation. Moreover, we speculate that the removal mechanism and chemical behaviour will be different at low and high Cr(vi) concentrations. In this study, the corm-straw was used to develop biochar (BC). nZVI was then loaded on this BC to synthesize nZVI–BC composite for the treatment of Cr(vi)-contaminated water. The effects of loading mass ratio of nZVI to BC, Cr(vi) initial concentration, dose of nZVI–BC composite, and pH on the remediation performance were studied. To specify the interfacial chemical behaviours and better describe the removal mechanism at different Cr(vi) initial concentrations, XRD and XPS analysis were conducted to detect crystal lattice and structural changes of the material before and after remediation at Cr(vi) initial concentrations of 20 mg L^−1^ and 100 mg L^−1^. The removal kinetics at Cr(vi) initial concentration of 20 mg L^−1^ and 100 mg L^−1^ were also simulated by pseudo first-order and pseudo second-order models to describe the key removal process, and the Langmuir and Freundlich modes were employed to fit the removal isotherm. We hope that our findings can provide a better choice for the utilization of corn straw as a resource in the HMs remediation.

## Experimental

### Materials

Biochar (BC) powder was synthesized from corn straw (composition is detailed in Table 1) using a stainless-steel furnace under the anaerobic condition at 550 °C followed by its sieving through a 200 Mesh Sieve. The synthesis process is detailed in our previous study.^41^ The potassium dichromate (K_2_CrO_4_) (AR, 99.7%), potassium tetrahydroborate (KBH_4_) (AR, 99.7%), iron(ii) sulfate heptahydrate (FeSO_4_·7H_2_O) (AR, 99.0%), sulfuric acid (H_2_SO_4_) (AR, 99.9%), acetone (AR, 99.9%) and other analytical chemical reagents were provided by Gansu Xingzhongsheng Trading Co., Ltd, Lanzhou, China. Ultrapure water was used in all sample preparation processes. Deoxygenated water and 99.99% pure nitrogen were used in the preparation of nZVI and nZVI–BC materials.

**Table 1:** Composition of the corn stalk used in the experiment

pH	EC (mS cm^−1^)	Total N (g kg^−1^)	Total K (%)	Total P (%)	Total C (g kg^−1^)	Fe	Cr
8.6	3.78	0.1441	17.3	0.189	6.7	0	0

### Preparation of nZVI and nZVI–BC composites

The removal agents (nZVI and nZVI–BC composite) were synthesized by liquid-phase reduction, as detailed in our previous studies.^42^ A certain amount of BC was stirred with 300 mL FeSO_4_·7H_2_O solution (0.12 mol L^−1^) for 15 minutes. Then, in this solution, NaBH solution (120 mL, 0.25 mol L^−1^) was added dropwise with continuous stirring for another 15 minutes after the complete reaction. Finally, the nano zero-valent iron-loaded on BC was obtained by filtering the reactant. The amounts of BC were decided by the mass ratio of iron to BC (*W*_Fe_ : *W*_BC_ = 1 : 3 and 1 : 5). The whole synthesis process was carried out under the protection of pure nitrogen (99.99%).

### Hexavalent chromium removal experiments

Cr(vi) solutions in the range of initial concentrations (20–100 mg L^−1^) were prepared by dissolving K_2_CrO_4_ in ultrapure water. Batch experiments were carried out by separately adding a certain amount of BC, nZVI–BC (*W*_Fe_ : *W*_BC_ = 1 : 3 and 1 : 5), and pure nZVI into 100 mL Cr(vi) solutions with different initial concentrations at room temperature (20 °C) and a thermostatic oscillator was used to keep the reaction complete. After 3 h, the solution was filtrated by a 0.45 μm filter and the Cr(vi) level was measured at 540 nm by employing a UV-Vis spectrophotometer (TU-1900PC, China). The pH value of the solutions was 6.0 ± 0.2.

The nZVI–BC dosage and Cr(vi) initial concentration effects were also studied. Added nZVI–BC composite with a dosage range of 1–8 g L^−1^ into 100 mL Cr(vi) solution (initial concentration:20–100 mg L^−1^) for oscillatory reaction (180 rpm), then the solution was filtrated by 0.45 μm filter after 3 h and the concentration of Cr(vi) was determined. The pH value of the solutions was 6.0 ± 0.2.

In addition, the effects of solution pH value on Cr(vi) remediation were investigated with an initial concentration of 100 mg L^−1^. The solution pH was adjusted by HCl (1 : 1) and NaOH (10 mol L^−1^) in the range of 2–10. The dose of nZVI–BC was 2.0 g L^−1^ and the volume of Cr(vi) solution was 100 mL. All the treatments have three repetitions. Removal efficiency (*E*, %) and capacity (*Q*, mg g^−1^) were calculated by the following formulas:1
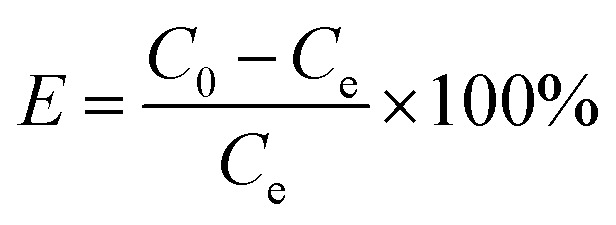
2
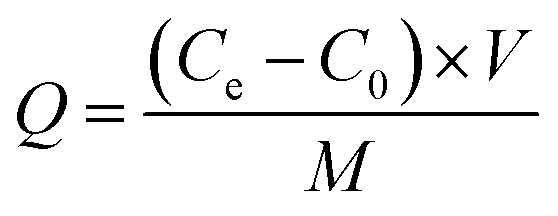
Here *C*_0_ and *C*_e_ were the Cr(vi) levels before and after reaction, mg L^−1^. V was the volume of Cr(vi) solution, mL. *M* was the mass of the added agents, g.

### Removal kinetics and isotherms

At room temperature, 1.0 g and 3.0 g nZVI–BC (*W*_Fe_ : *W*_BC_ = 1 : 3) were separately added to 500 mL Cr(vi) solution (20 mg L^−1^ and 100 mg L^−1^) for oscillatory reaction (180 rpm). At regular intervals (5 min, 10 min, 15 min, 20 min, 30 min, 45 min, 60 min, 90 min, 120 min, 180 min), a certain volume of the solution was taken by 0.45 microns-filter and the Cr(vi) concentration was determined. Each sample was repeated three times.

### Characterization

The BC, nZVI, and nZVI–BC powders were scanned by TEM-EDS ((JEM-1230, JEOL, Japan) to observe their morphology and surface textures before reaction with Cr(vi). The XRD (Ultima IV, Japan) was conducted to determine and observe the iron phase crystallographic structure of these agents (BC, nZVI, and nZVI–BC). The nZVI–BC samples before and after reacting with Cr(vi) solutions (20 mg L^−1^ and 100 mg L^−1^) at a dosage of 2 g L^−1^ and 6 g L^−1^ were used for XPS (AXIS Supra, Japan) analysis.

## Results and discussion

### Morphology analysis of the prepared materials (TEM and EDS analysis)

**Fig. 1:**
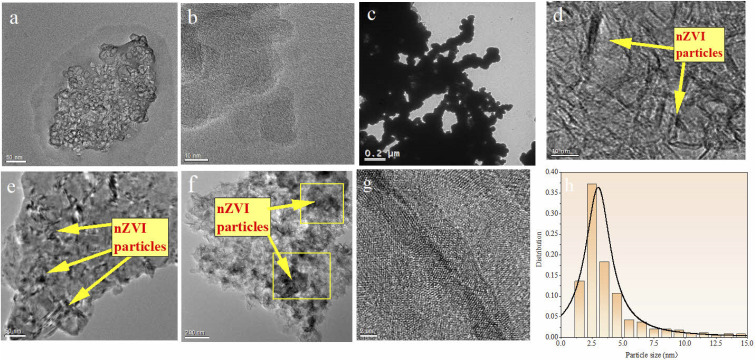
(a and b) TEM micrographs of biochar (BC); (c) TEM micrographs of nano zero-valent iron (nZVI); (d–f) TEM micrographs of nZVI–BC (*W*_Fe_ : *W*_BC_ = 1 : 3) materials; (g) HR-TEM images of nZVI–BC biochar; (h) size distribution curve of nZVI particles on BC surface.

**Fig. 2:**
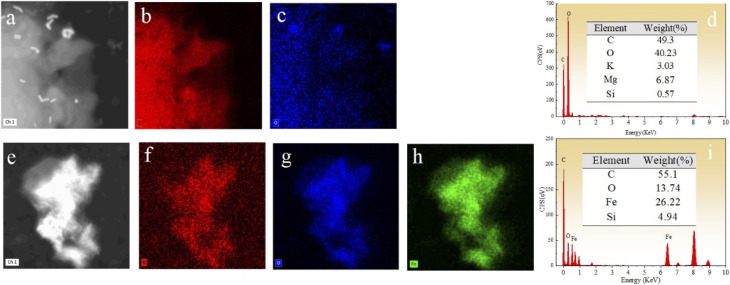
(a–d) EDS images of prepared biochar (BC); (e–i) EDS images of prepared nZVI–BC (*W*_Fe_ : *W*_BC_ = 1 : 3) materials.

The morphology of BC, nZVI, and nZVI–BC was studied by TEM analysis (Fig. 1). TEM images show that BC had a porous structure and huge surface area (Fig. 1a and b). Compared to bare nZVI particles which aggregated together mostly (Fig. 1c), the iron particles loaded on BC had a smaller size which are dispersed on the BC surface and some of them deposited into the lumen of BC and caused the lumen blocking and the surface area of nZVI–BC reached 175 m^2^ g^−1^ (Fig. 1d–f). The crystalline property of nZVI loaded on BC was also investigated by using HR-TEM (Fig. 1h). The size distribution curve of the nZVI particle loaded on BC (Fig. 1i) was analyzed by computer program known as ImageJ. The results have shown that the minimum particle size of nZVI was 2.945 nm, and the average size was 5.108 nm. But it also can be found that the nZVI particles with 100 nm were distributed on the BC surface. The EDS analysis was then employed to characterize the composite better and the results were shown in Fig. 2. The EDS spectrum (Fig. 2a–d) displayed that the corn-straw BC was mainly composed of C and O (w, %: 49.3, 40.23) and a few other elements such as K, Mg, and Si (w, %: 3.03, 6.87, 0.57). The EDS morphology of nZVI–BC changed a lot as shown in Fig. 2e–i. The images showed the existence of Fe along with C and O elements. The amounts of C, Fe, and O were respectively 55.1%, 13.74%, and 26.22%. This indicated that Fe was loaded on the BC.

### Cr(vi) removal performance

**Fig. 3:**
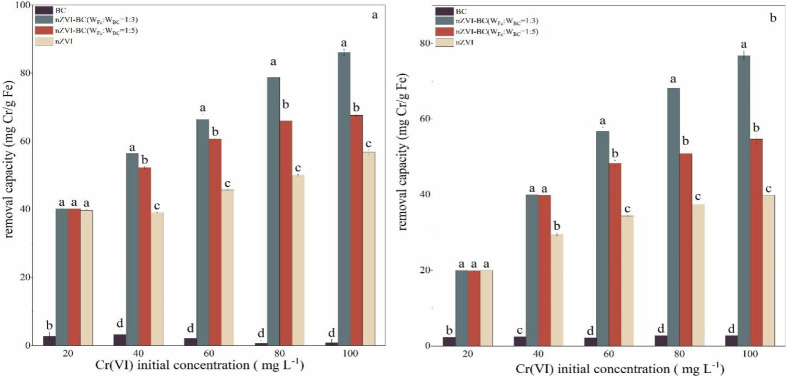
Comparison of removal capacity of different adsorbents addition: (a) net iron addition: 0.5 g L^−1^; (b) net iron addition: 1.0 g L^−1^; the Cr(vi) solution volume: 100 mL; initial pH value: 6.0 ± 0.2.

Cr(vi) removal performance of the modified material was studied by comparing the removal efficiency of BC, nZVI, and nZVI–BC (*W*_Fe_ : *W*_BC_ = 1 : 3 and 1 : 5)) (Fig. 3). Compared with the other three materials, the BC had little effect on the removal of Cr(vi). At Cr(vi) concentration of 20 mg L^−1^, there was no significant difference in removal capacity between pure nZVI and nZVI–BC. This can be linked to the lower concentration of Cr(vi) which was effectively removed by nZVI either alone or loaded on BC. However, with an increase in the concentration of Cr(vi), difference between the efficiency of bare nZVI and nZVI/BC became evident. When the initial concentration of Cr(vi) was 100 mg L^−1^, the removal capacity increased to 56.77 mg Cr per g Fe (nZVI), 86.13 mg Cr per g Fe (nZVI–BC (*W*_Fe_ : *W*_BC_ = 1 : 3)), and 67.61 mg Cr per g Fe (nZVI–BC (*W*_Fe_ : *W*_BC_ = 1 : 5)) at a net iron dosage of 0.5 g (Fig. 3a), and at a net iron dosage of 1.0 g (Fig. 3b), the value increased to 39.80 mg Cr per g Fe (nZVI), 63.59 mg Cr per g Fe (nZVI–BC (*W*_Fe_ : *W*_BC_ = 1 : 3)), and 54.67 mg Cr per g Fe (nZVI–BC (*W*_Fe_ : *W*_BC_ = 1 : 5)).

These results show that loading nZVI on BC can highly ameliorate its Cr(vi) remediation performance and the load ratio between nZVI and BC also affected the remediation capacity. It can be found that nZVI–BC (*W*_Fe_ : *W*_BC_ = 1 : 3) had a better removal performance than nZVI–BC (1 : 5) (*p* < 0.05). The BC played the role of dispersion carrier more, while the nZVI provided exchangeable electrons for the conversion of Cr(vi) to Cr(iii) and contributed to higher removal performance of Cr(vi). The decrease in the ratio of iron to carbon significantly led to the reduction of electron donors which decreased the Cr(vi) removal capacity and efficiency.

### Effects of nZVI–BC dosage and Cr(vi) concentration

**Fig. 4:**
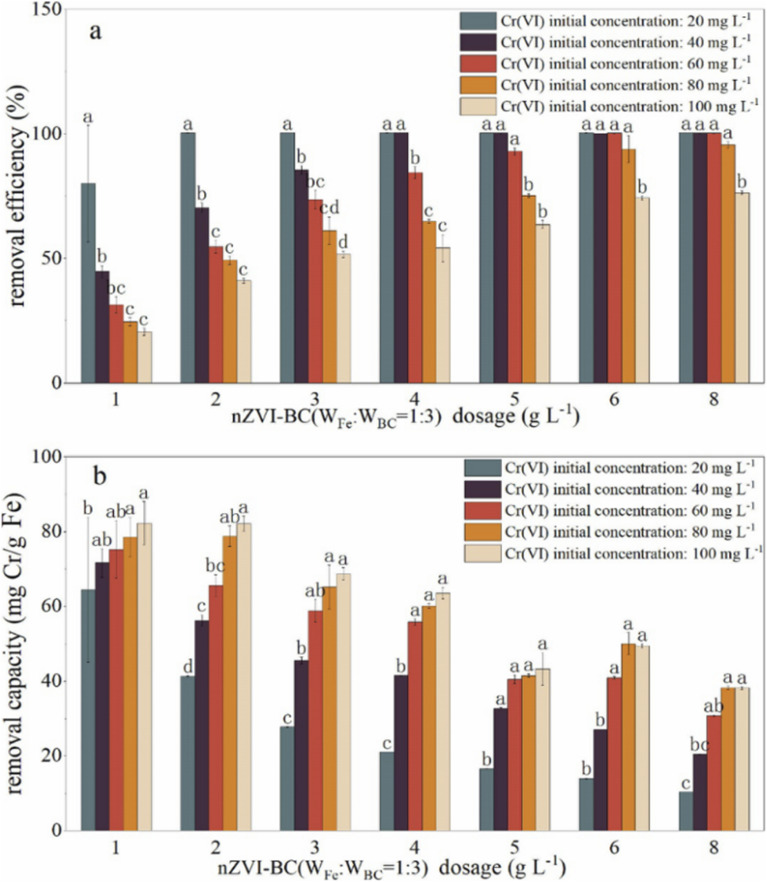
The effect of nZVI–BC (*W*_Fe_ : *W*_BC_ = 1 : 3) dosage and Cr(vi) initial concentration on: (a) removal efficiency; (b) removal capacity (the Cr(vi) solution volume: 100 mL; initial pH value: 6.0 ± 0.2; (a–d) means the significance difference level).

The effects of nZVI–BC composite dosage and Cr(vi) level on Cr(vi) remediation efficiency were studied to obtain the optimum experiment conditions (Fig. 4). The removal efficiency raised significantly with nZVI–BC dosage increase (*P* < 0.05) and dropped significantly with Cr(vi) concentration increase (*P* < 0.05) (Fig. 4a). At low Cr(vi) concentration (20 mg L^−1^), even though the removal efficiency is high, the removal efficiency still increased from 79.98% to 100% by increasing the nZVI–BC dosage from 1 g L^−1^ to 2 g L^−1^. While at high Cr(vi) concentration (100 mg L^−1^), the removal efficiency increased from 20.56% to 74.20% by increasing nZVI–BC dosage from 1 g L^−1^ to 6 g L^−1^, and further increase of nZVI–BC showed only a plateau trend to 76.37%. It also demonstrated in Fig. 4a that removal efficiency dropped significantly with an increase in Cr(vi) concentration (*P* < 0.05). With Cr(vi) concentration increasing in a range of 20–100 mg L^−1^, the removal efficiency decreased sharply from 79.98% to 20.56% at the low amount of the composites (1 g L^−1^), while it dropped from 100% to 76.37% at high composites amount (8 g L^−1^). The improvement in remediation according to the nZVI–BC dosage increase was due to the complex removal process containing adsorption and oxidation–reduction simultaneously which have been pointed out in this study. The increase of nZVI–BC dosage represented a larger available surface area which can provide more free adsorption sites and more reductive which can increase and promote the occurrence of redox. When both the surface and solution concentration of Cr(vi) settle to equilibrium, the increase of removal turned to slow down.

However, it can be illustrated in Fig. 4b that the removal capacity decreased with an increase in the composite dosage and increased with raising the Cr(vi) concentration (*P* < 0.05). With composite dosage increasing in a range of 1–8 g L^−1^, the removal capacity decreased from 64.47 mg Cr per g Fe to 10.38 mg Cr per g Fe at a concentration of 20 mg L^−1^ and decreased from 82.24 mg Cr per g Fe to 38.18 mg Cr per g Fe at a concentration of 20 mg L^−1^. When Cr(vi) concentration increased from 20 mg L^−1^ to 100 mg L^−1^, the removal capacity increased in a range of 64.46–84.24 mg Cr per g Fe at a dose of 1 g L^−1^ and in a range of 10.38–38.18 Cr per g Fe at a dose of 8 g L^−1^. This may be because there are large amounts of active sites on synthesized material, even a small addition can arrive at high removal efficiency. Because of the removal process accompanied by the adsorption and oxidation–reduction, and there was a maximum adsorption capacity for Cr(vi), once the adsorption capacity approached saturation, it will no longer increase even if the hexavalent chromium content increase.

Therefore, the 2 g L^−1^ and 6 g L^−1^ were considered to be the optimum dose at Cr(vi) concentrations of 20 mg L^−1^ and 100 mg L^−1^, respectively.

### Effect of initial pH on remediation performance

**Fig. 5:**
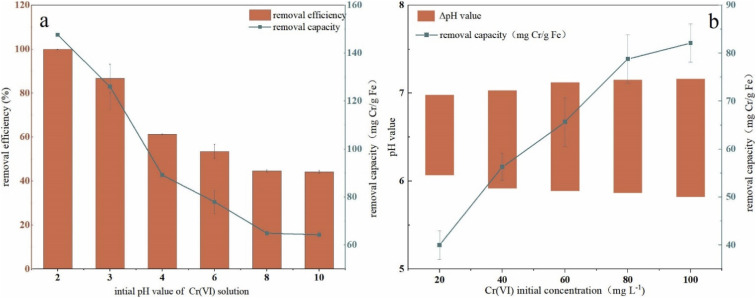
(a) Effect of pH on Cr(vi) remediation (Cr(vi) concentration: 100 mg L^−1^; nZVI–BC (*W*_Fe_ : *W*_BC_ = 1 : 3) dosage: 2 g L^−1^; Cr(vi) solution volume: 100 mL); (b) pH value change in remediation process (nZVI–BC (*W*_Fe_ : *W*_BC_ = 1 : 3) dosage: 2 g L^−1^; Cr(vi) solution volume: 100 mL).

To investigate the effect of the initial pH value of Cr(vi) solution on remediation, pH was adjusted in a range of 2–10 and the results were presented in Fig. 5a. It can be seen that with an increase in solutions pH, the removal efficiency first dropped rapidly from 100% to 61.3% in the range of 2–4, and the decrease turned to be slower in the range of 4–8 (from 61.32% to 44.59%). When the pH value is beyond 8, the removal efficiency kept in a plateau trend and finally decreased to 44% (44.60% at pH 8, 44.17% at pH 10). Removal capacity had the same trend to efficiency with pH increase, which got the maximum value of 147.59 mg Cr per g Fe at pH 2 and a minimum value of 64.24 mg Cr per g Fe at pH 10. The pH change in solution (ΔpH, ΔpH = pH value_after reaction_ − pH value_before reaction_) in the remediation process were shown in Fig. 5b. As it was shown, the pH value of the solution increased to a range of 7.0–7.25 after remediation and the highest initial concentration (100 mg L^−1^) corresponded to the maximum ΔpH which almost arrived at 1.5. These results indicated that H^+^ took part in the remediation process and was expended during the reduction of Cr(vi). The H^+^ consumption due to Cr(vi) reduction led to an increase of OH^−^ in the solution.

The performance of nZVI–BC affected by pH may be due to many reasons because the process was complex and influenced by many factors such as nZVI activity, species of Cr(vi), and surface charges of BC. A previous study proved that at low pH, the Cr remediation was an acid-driven surface-mediated process.^43^ Cr(vi) mainly existed in the form of HCrO_4_^−^ in a pH range of 1–6, in the form of Cr_2_O_7_^2−^ with a pH range of 6.0–7.5, and in the form of CrO_4_^2−^ when pH was beyond 7. Due to the adsorption free energy of the HCrO_4_^−^ being higher than that of the CrO_4_^2−^, the HCrO_4_^−^ was easier to be adsorbed than CrO_4_^2−^ which led to a higher removal efficiency and capacity. Some studies also revealed that low pH can cause a more positive charge on the BC surface which also improved the remediation.^44^ Another reason may be that there existed an oxide layer on the surface which blocked the active reaction sites of nZVI.^45^ At an acidic pH, the oxide layer was cleared and the blocked active reaction sites were released which improved the reduction of Cr(vi) leading to a high removal performance.

### Removal kinetics and isotherms

To further study the remediation mechanism and investigate rate-controlling steps, the removal kinetics and isotherms studies have been done. For kinetic studies, the effect of contact time on Cr(vi) removal was investigated in 100 mL solutions at two concentrations (20 mg L^−1^ and 100 mg L^−1^) with the agent dose of 2 g L^−1^ and 6 g L^−1^. The experiment data were fitted by the nonlinear pseudo-first-order (PFO) and pseudo-second-order (PSO) model (formula (3) and (4)). For isotherm studies, the experiments were carried out at a pH value of 6.0 ± 0.2, the reaction time of 3 h, the temperature of 293 K, and agents dose level of 2 g L^−1^ and 6 g L^−1^. The data were fitted by the nonlinear Langmuir and Freundlich model (formula (5) and (6)).

3 ln(*q*_e_ − *q*_*t*_) = ln *q*_e_ − *k*_1_*t*

4
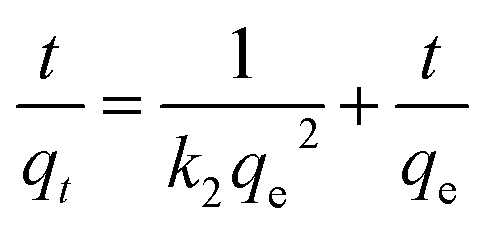
Here, *q*_e_ and *q*_*t*_ respectively mean the removal capacity at equilibrium time and time *t*, mg g^−1^; *k*_1_ was the first-order rate constant, min^−1^; *k*_2_ was the second-order rate constant, mg g^−1^ min^−1/2^;5
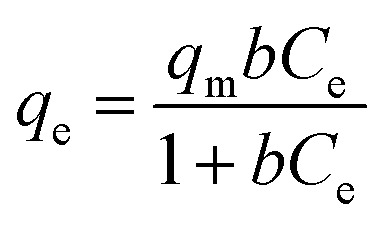

6 *q*_e_ = *k*_3_*C*_e_^1/*n*^

Here, *q*_e_ means the removal capacity at the initial concentration of *c*_e_, mg g^−1^; *c*_e_ was the Cr(vi) initial concentration, mg L^−1^; *b* was the Langmuir constant and *k*_3_ was the Freundlich constant; *n* is the heterogeneity factor.

**Fig. 6:**
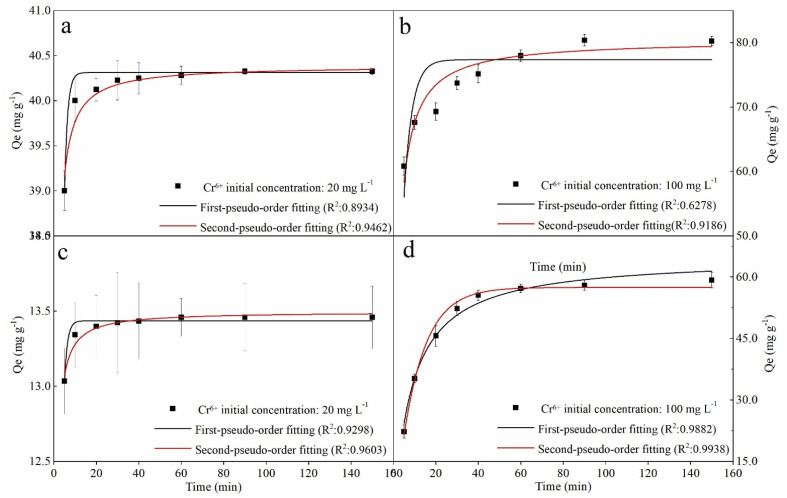
Kinetics fitting results of Cr(vi) removal: (a and b) nZVI–BC (*W*_Fe_ : *W*_BC_ = 1 : 3) dosage: 2 g L^−1^; (c and d) nZVI–BC (*W*_Fe_ : *W*_BC_ = 1 : 3) dosage: 6 g L^−1^ (pH value: 6.0 ± 0.2) (solution volume: 100 mL; pH value: 6.0 ± 0.2).

To the kinetics, the removal curves and fitting results were shown in Fig. 6 and Table 2. As can be seen in Fig. 6, the removal curves with an initial concentration of 20 mg L^−1^ showed a sharp rise in the first 20 min and then a flat (Fig. 6a and c), while the curves with an initial concentration of 100 mg L^−1^ showed rapid rising in the first 40 min (Fig. 6b and d). This can be attributed to the availability of mounts of vacant active sites on the fresh nZVI–BC surface, and then the pore diffusion of Cr(vi) slowed which led to the decrease in removal rate.^46^ For the fitting results, the *q*_e_ value calculated by PFO and PSO models all exhibited well-agreement with the experiment value. But based on the correlation coefficient value (*R*^2^), the PSO (0.9462, 0.9603, 0.9186, 0.9938) gave a better fitting at the four different initial conditions. PSO model was based on the solid adsorption capacity and the results demonstrated that the remediation process was dominated by the chemical adsorption.^47^ Moreover, it also can be found in Fig. 6b and d that the real data points show a step change between 20 min and 40 min at an Cr(vi) initial concentration of 100 mg L^−1^ which cannot be well captured by the proposed adsorption model, especially with a low agent dose. It can be speculated that the adsorption of Cr(vi) at a high concentration was not only electrostatic adsorption between nZVI and Cr(vi). Based on our data and the previous study,^48^ there exist three steps in the adsorption process including (i) due to the superior surface activity of the agents, it was mainly the external surface or instantaneous adsorption during the first 20 min; (ii) the next step between 20 min to 40 min was the gradual adsorption stage where occurred pore diffusion; and, (iii) the last stage was attributed to the final adsorption equilibrium. At an initial Cr(vi) concentration of 100 mg L^−1^, active electrons on the surface of the agent were not enough because of the low agent dose (2 g L^−1^). Therefore, the pore diffusion became rate limiting step which led to a lower removal capacity of the agents compared to the calculated value by the proposed adsorption model.

**Table 2:** Kinetic parameters for pseudo-first-order (PFO) and pseudo-second-order (PSO) models (*Q*_e,exp_: experimental removal capacity; *Q*_e,cal_: calculated removal capacity)

nZVI–BC (*W*_Fe_ : *W*_BC_ = 1 : 3) dosage (g L^−1^)	Cr(vi) concentration (mg L^−1^)	*Q*_e,exp_ (mg g^−1^)	Pseudo-first order			Pseudo-second order		
			*Q*_e,cal_ (mg g^−1^)	*k*_1_ (min^−1^)	*R* ^2^	*Q*_e,cal_ (mg g^−1^)	*k*_2_ (mg g^−1^ min^−1/2^)	*R* ^2^
2	20	40.32	40.31	0.70	0.9418	40.39	0.15	0.9462
	100	80.25	77.35	0.26	0.6278	80.41	0.006	0.9186
6	20	13.45	13.43	0.70	0.9298	13.50	0.45	0.9603
	100	59.21	57.55	0.09	0.9868	64.51	0.002	0.9862

**Fig. 7:**
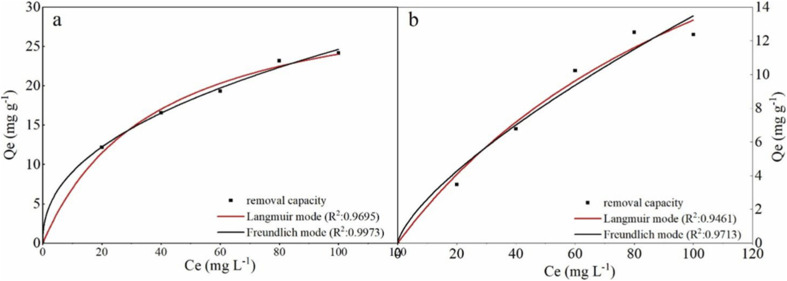
Isotherms fitting results of Cr(vi) removal: (a) nZVI–BC (*W*_Fe_ : *W*_BC_ = 1 : 3) dosage: 2 g L^−1^; (b) nZVI–BC (*W*_Fe_ : *W*_BC_ = 1 : 3) dosage: 6 g L^−1^ (solution volume: 100 mL; pH value: 6.0 ± 0.2).

To the isotherms, the removal curves and fitting results were shown in Fig. 7 and Table 3. The nZVI–BC composite removal capacity increased rapidly and then slowed down at a lower nZVI–BC dosage of 2 g L^−1^. While the dosage was increased to 6 g L^−1^, the removal capacity further increased. And it also can be seen that the fitting results were more consistent with the Freundlich model (0.9973, 0.9713). In previous studies, the Langmuir model was proposed based on the theory that all the sites of absorbent have an equal affinity toward the ions and the ions may form a monolayer coat on the surface of the absorbents, while the Freundlich model based on the equilibrium relationship between inhomogeneous surface and suggested an inhomogeneous system.^49,50^ Combined with the fitting results, we can concluded that the adsorption of hexavalent chromium was a multi-molecular layer adsorption process and parts of the adsorbed Cr(vi) ions were tightly bonded to the nZVI–BC surface and were not readily desorbable.^48^

**Table 3:** Parameters for Langmuir and Freundlich models (*Q*_e,exp_: experimental removal capacity; *Q*_e,cal_: calculated removal capacity)

nZVI–BC (*W*_Fe_ : *W*_BC_ = 1 : 3) dosage (g L^−1^)	*Q*_e,exp_ (mg.g^−1^)	Langmuir			Freundlich			
		*Q*_m,cal_ (mg g^−1^)	*b* (L mg^−1^)	*R* ^2^	*k*_3_ (L mg^−1^)	*n*	1/*n*	*R* ^2^
2	20.53	33.09	0.03	0.9695	3.31	2.30	0.43	0.9973
6	12.09	30.25	0.01	0.9461	0.50	1.40	0.71	0.9713

### Interfacial chemical behaviours in the remediation of hexavalent chromium

**Fig. 8:**
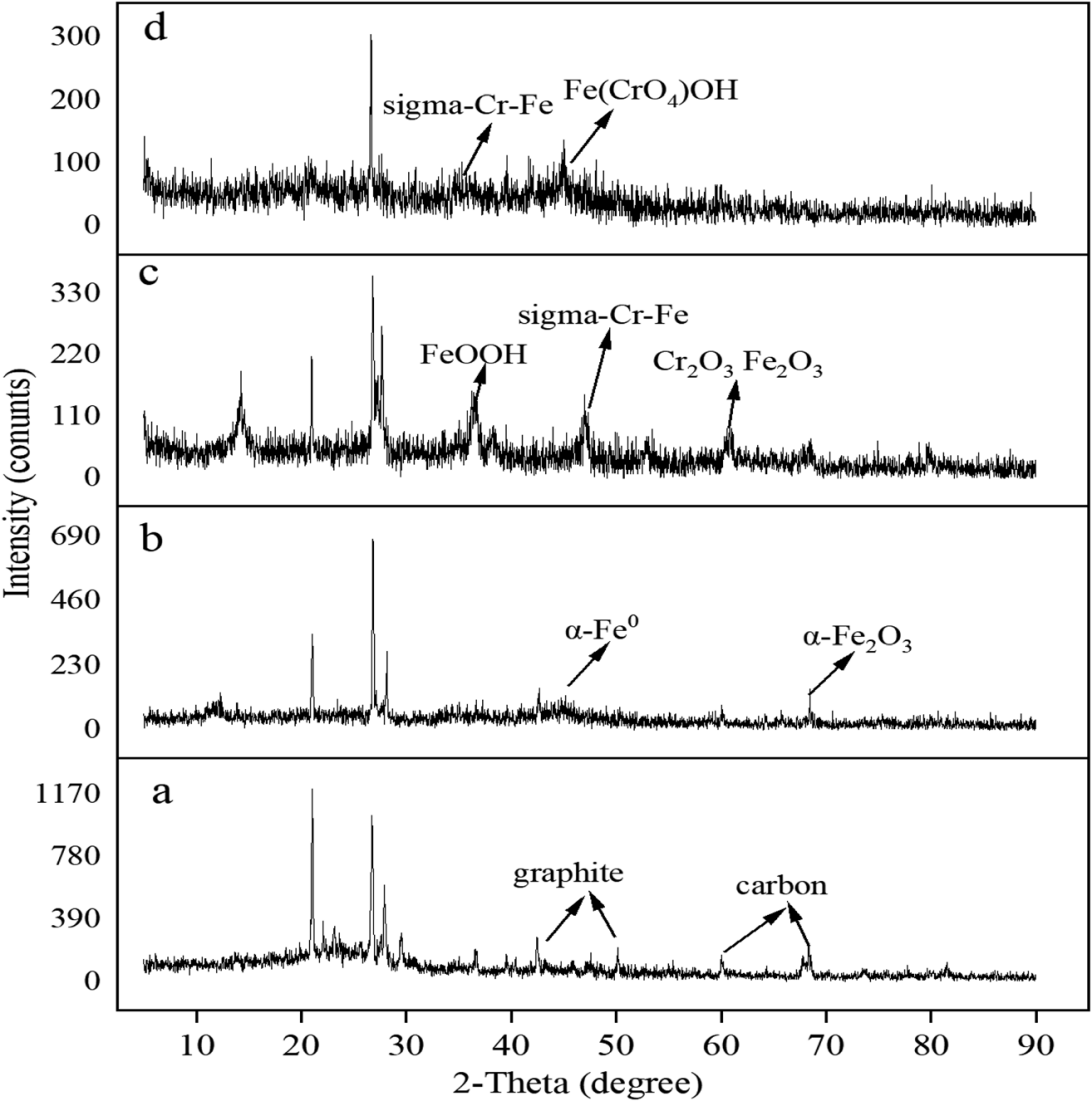
XRD scan of the material (a) BC; (b) nZVI–BC (*W*_Fe_ : *W*_BC_ = 1 : 3) composite; (c) nZVI–BC (*W*_Fe_ : *W*_BC_ = 1 : 3) composite after reaction with Cr(vi) at the concentration of 20 mg L^−1^; (d) nZVI–BC (*W*_Fe_ : *W*_BC_ = 1 : 3) composite after reaction with Cr(vi) at the concentration of 100 mg L^−1^ (dosage of nZVI–BC (*W*_Fe_ : *W*_BC_ = 1 : 3): 2 g L^−1^; the Cr(vi) solution volume: 100 mL; initial pH value: 6.0 ± 0.2).

**Fig. 9:**
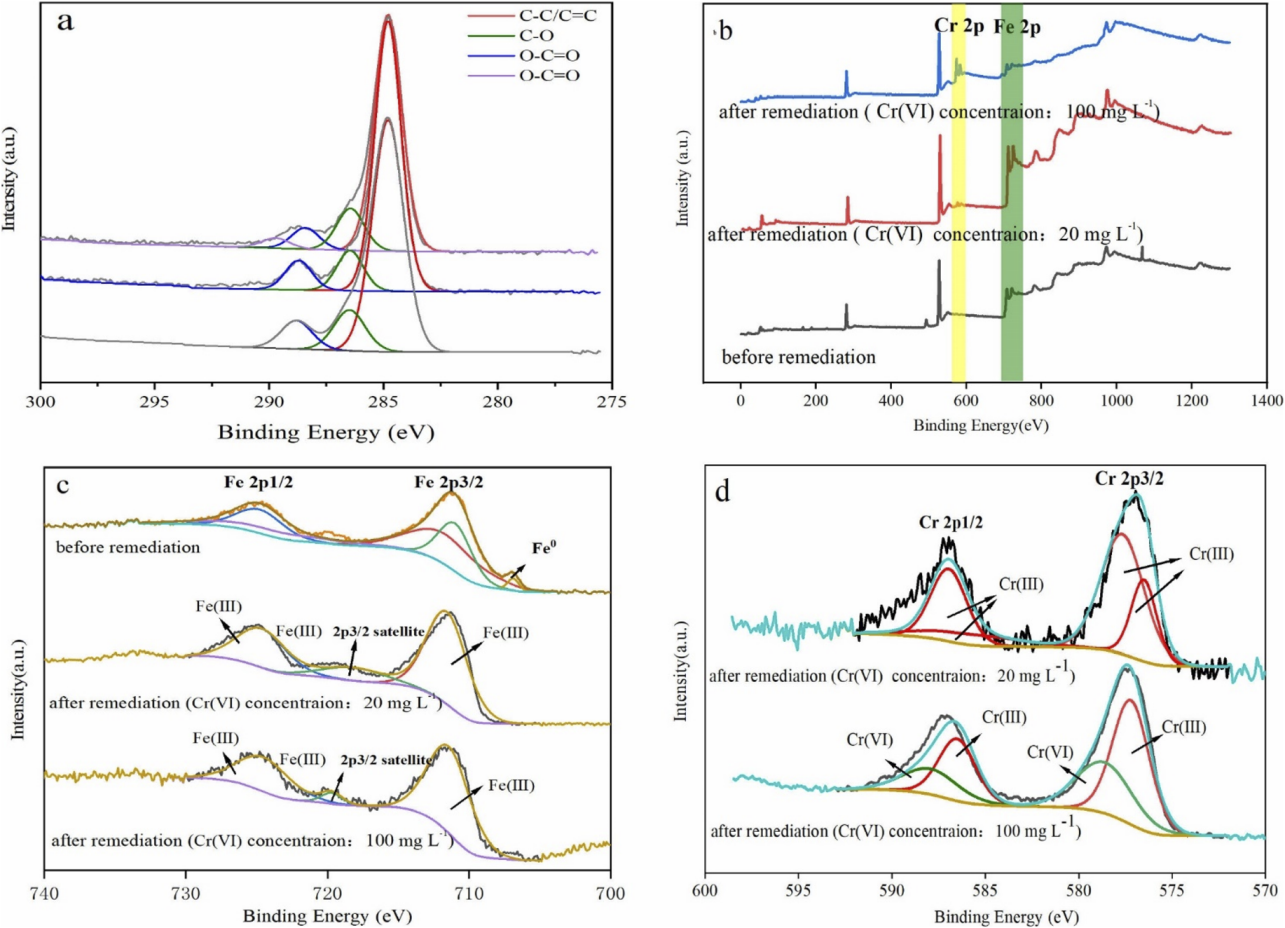
XPS scan of nZVI–BC (*W*_Fe_ : *W*_BC_ = 1 : 3) composites before and after reaction with different concentration of hexavalent chromium: (a) C 1s scan; (b) full scan; (c) Fe 2p high spectral scan; (d) Cr 2p high spectral scan (dosage of nZVI–BC (*W*_Fe_ : *W*_BC_ = 1 : 3) 2 g L^−1^; the Cr(vi) solution volume: 100 mL; initial pH value: 6.0 ± 0.2).

To investigate the surface chemical behaviour, XRD and XPS were used to determine the composition, structure, and chemical or elemental composition of the agent surface during the remediation process (Fig. 8 and 9). Compared to the XRD scan of the BC sample (Fig. 8a), in which the several peaks at 21°, 26.7°, 41.5°, 68.3° represented different crystals of carbon, the peaks at 44.7°, 68.4° in Fig. 8b represented α-Fe^0^ (ref. 51) and this unambiguously proved that the iron was loaded successfully and it was further confirmed by the XPS results. After reaction with Cr(vi), the XRD patterns of nZVI–BC were shown in Fig. 8c and d. The peaks were respectively matched with different forms of iron-chromium oxide, including FeOOH (peaks at 36.2°, 60.5°), sigma Cr–Fe (peaks at 47°, 80.2°), Cr_2_O_3_. Fe_2_O_3_ (peaks at 60.5°) at Cr(vi) initial concentration of 20 mg L^−1^ in Fig. 8c. To the XRD pattern at Cr(vi) initial concentration of 100 mg L^−1^ in Fig. 8d, the main iron-chromium was sigma Cr–Fe (peak at 35.3°) and Fe(CrO_4_)OH (peaks at 47°). These results indicated that there happened a reduction–oxidation reaction between Cr(vi) and Fe^0^.

The XPS results of the nZVI–BC composite before and after reaction with Cr(vi) were shown in Fig. 9. The four peaks in Fig. 9a at 284.8 eV, 286.45 eV, 288.8 eV, and 289.83 eV respectively corresponded to carbon which existed in the forms of C–C/C=C (284.8 eV), C–O (286.45 eV), O–C=O (288.8 eV)^52^ before reaction. After remediation, the peak at 289.83 eV disappeared and the content of the C=O weakly increased from 7.14% to 8.61% (Cr(vi) initial concentration: 20 mg L^−1^) and 9.47% ((Cr(vi) initial concentration: 100 mg L^−1^). This may be due to the synthesis process, C–O–Fe formed as Fe soaked with BC by ligand exchange, chelating, or bridging. When in reaction with Cr(vi), the breakage of the O–Fe bond which was caused by the electron transferring between Cr–Fe during the reduction led to the forming of C–C=O bonds or iron oxides.^53^

The full scans of nZVI–BC composites before and after the reaction were shown in Fig. 9b. The double-peak at 560 eV and 600 eV which appeared after reaction corresponded to Cr(vi) and represented that Cr(vi) was adsorbed onto the surface of the nZVI–BC composite.

The Fe 2p high spectral scan was shown in Fig. 9c. Before reaction, the peak at 706.88 eV corresponded to Fe^0^ 2p3/2,^54^ which indicated that the nZVI was successfully loaded onto the BC surface. The peaks at 710.97 eV and 711.68 eV respectively marked the existence of Fe^2+^ and Fe^3+^ and this suggested that zero-valent iron was oxidized partially in the process of synthesis. After reaction, the double peaks at 711.56 eV, 724.66 eV (Cr(vi) initial level: 20 mg L^−1^) and 711.41 eV, 724.51 eV (Cr(vi) initial level: 100 mg L^−1^) respectively corresponded to Fe^3+^. The peaks at 718.19 eV and 719.68 eV were their satellite peak. The Cr 2p high spectral scan was shown in Fig. 9d. On the spectra of 20 mg L^−1^ Cr(vi) initial concentration, the double peaks at 577.66 eV, 576.50 eV, and 586.96 eV, 585.80 eV were respectively corresponded to Cr^3+^ 2P_3/2_ and Cr^3+^ 2P_1/2_ (ref. 55) which indicated that the Cr(vi) was completely reduced into Cr(iii). While on the spectra of 100 mg L^−1^ Cr(vi) initial concentration, the peaks at 577.20 eV and 586.50 eV were ascribed to Cr^3+^ 2P_3/2_ and Cr^3+^ 2P_1/2_, and the peaks at 578.63 eV and 587.93 eV were assigned to Cr^6+^ 2P_3/2_ and Cr^6+^ 2P_1/2_.^56^ The amounts percentages of Cr(iii) and Cr(vi) were 61.46% and 38.54%. The results indicated that part of Cr(vi) was reduced to Cr(iii) and the rest of Cr(vi) was adsorbed onto the composite surface at high Cr(vi) concentration, while Cr(vi) was completely reduced at low Cr(vi) concentration.

**Fig. 10:**
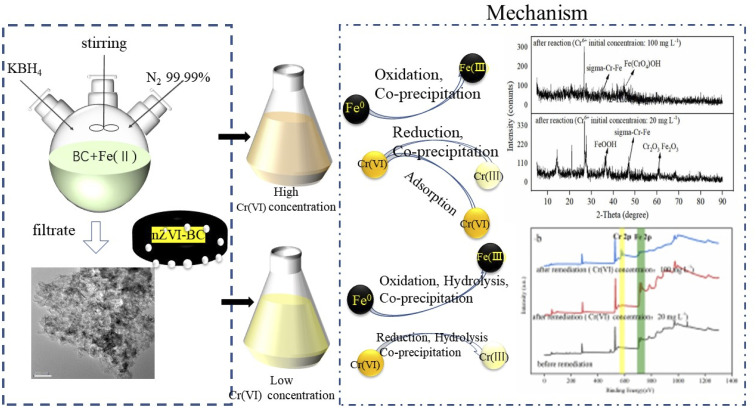
Mechanism of Cr(vi) removal by nZVI–BC in aqueous medium.

By these results, it can be concluded that on the surface of the nZVI–BC composite there happened adsorption, chemical reduction–oxidation reaction, and co-precipitation in the remediation process and the removal mechanism was shown in Fig. 10. Firstly, BC exhibited its strong adsorption properties and Cr(vi) was adsorbed onto the nZVI–BC composite surface. Then occurred reduction–oxidation. The Fe^0^ and Fe^2+^ played as electron donors, Cr(vi) as the electron acceptors and BC played as the electron transfer medium. Fe^0^/Fe^2+^ lost electrons and exited in the form of Fe^3+^, while Cr_2_O_7_^2−^ transformed into the form of Cr_2_O_4_^2−^ because of obtaining electrons and then produced the Cr–Fe oxide (formula (7)–(9)).^57^ Combined the results of XRD analysis, FeOOH, and Cr_2_O_3_·Fe_2_O_3_ existed on the nZVI–BC surface at the same time with Cr(vi) level of 20 mg L^−1^, while with Cr(vi) level of 100 mg L^−1^, there still existed Cr(vi) in form of Fe(CrO_4_)OH. Therefore, we hypothesized that at a low Cr(vi) level, due to the excessive reductive, the Fe^0^ reacted with water and formed the OH^−^ which promoted the hydrolysis of the generated Fe^3+^ and Cr^3+^ (formula (10)–(12)),^23^ then occurred the co-precipitation between the production of hydrolysis. At the same time, the Fe^2+^ also reacted with OH^−^ and generated FeOOH (formula (13)). At Cr(vi) initial level of 100 mg L^−1^, parts of the adsorbed Cr(vi) took part in the chemical reduction–oxidation reaction (formula (7)–(9)), while the excessive Cr(vi) reacted with Fe^3+^ (formula (14)) and precipitated on BC surface because of the excellent adsorption capacity and porosity of BC. And the hypothesis was perfect consistent with our experiment results.

In conclusion, there occurred adsorption, reduction–oxidation reaction, and co-precipitation on the surface of the nZVI–BC composite. The BC not only played as the carrier to disperse and stabilize nZVI but also as the adsorbent and medium of electron transfer between nZVI and BC because of its excellent electrical conductivity.^36^7
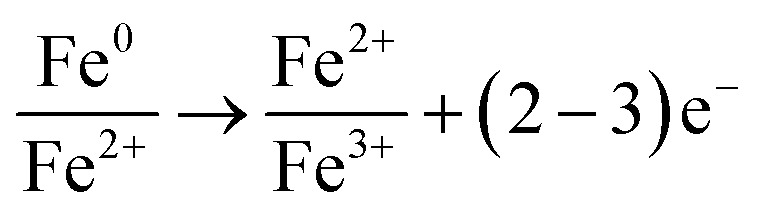

8 Cr_2_O_7_^2−^ + 3H^+^ + 6e^−^ → Cr_2_O_4_^2−^ + 3H_2_O

9 *x*Cr^3+^ + (1 − *x*)Fe^3+^ + 3H_2_O → (Cr_*x*_Fe_1−*x*_)(OH)_3_ + 3H^+^

10 Fe^0^ + H_2_O → Fe^2+^ + H_2_↑ + 2OH^−^

11 2Fe^3+^ + 6OH^−^ ↔ Fe(OH)_3_(s) ↔ Fe_2_O_3_(s) + 3H_2_O

12 2Cr^3+^ + 6OH^−^ ↔ Cr(OH)_3_(s) ↔ Cr_2_O_3_(s) + 3H_2_O

13 Fe^2+^ + O_2_↑ + 8OH^−^ → 4FeOOH + 2H_2_O

14 Fe^3+^ + HCrO_4_^−^ + 2OH^−^ → Fe(CrO_4_)OH + H_2_O

## Conclusions

Using corn-straw biochar as load material, the nZVI–BC composite was prepared by reduction reaction. TEM-EDS image proved that loading nZVI on BC can highly solve its agglomeration. The remediation experiments of Cr(vi) solution with nZVI–BC composite were carried out and the results revealed that the remediation efficiency and capacity of Cr(vi) can be efficiently improved by loading nano zero-valent iron on biochar, and the removal efficiency and capacity were also influenced by the amount of the composite, Cr(vi) concentration and solution pH. Increasing composite amounts can significantly increase removal efficiency, while the capacity decreased according to the composite dosage increase. The removal efficiency and capacity reached the maximum value at low pH and the pH value of Cr(vi) solution increased significantly after remediation. Chemical behaviour on the nZVI–BC surface was evaluated by the XRD and XPS analyses which included adsorption, chemical reduction–oxidation reaction, and co-precipitation in the remediation process of Cr(vi). The Cr(vi) was adsorbed by BC on its surface and then gained the electron from Fe^0^ which played as the electron donor and transformed it into Cr(iii) and then formed stable Cr–Fe oxide (FeCr_2_O_4_). The hydrolysis of Fe(iii) and Cr(iii) occurred simultaneously at low Cr(vi) levels due to the excessive Fe^0^ and products like Cr_2_O_3_ and Fe_2_O_3_ were also precipitated on its surface. The removal kinetics and isotherms were also studied. The PSO and Freundlich models were more consistent with the fitting results, and this revealed that the removal process was dominated by the chemical reduction and the adsorption process was a multi-molecular layer adsorption.

## Author contributions

Yuzhen Wei: conceptualization, methodology, formal analysis, writing–original draft; Run Chu: writing – review & editing; Qinhu Zhang: data management; Muhammad Usman: writing – review & editing; Fasih Ullah Haider: writing – review & editing; Liqun Cai: supervision and project administration.

## Conflicts of interest

The authors declare that this work is original and has not been published elsewhere, nor has been submitted to any other journal and all the authors have agreed to submit the manuscript in ‘RSC Advances’. Also, we declare that there are no conflicts of interest.

## Supplementary Material
